# Simian Immunodeficiency Virus (SIV)-Specific Chimeric Antigen Receptor-T Cells Engineered to Target B Cell Follicles and Suppress SIV Replication

**DOI:** 10.3389/fimmu.2018.00492

**Published:** 2018-03-20

**Authors:** Kumudhini Preethi Haran, Agnes Hajduczki, Mary S. Pampusch, Gwantwa Mwakalundwa, Diego A. Vargas-Inchaustegui, Eva G. Rakasz, Elizabeth Connick, Edward A. Berger, Pamela J. Skinner

**Affiliations:** ^1^Department of Veterinary and Biomedical Sciences, University of Minnesota, St. Paul, MN, United States; ^2^Laboratory of Viral Diseases, National Institute of Allergy and Infectious Diseases, National Institutes of Health, Bethesda, MD, United States; ^3^Wisconsin National Primate Research Center, University of Wisconsin-Madison, Madison, WI, United States; ^4^Division of Infectious Diseases, University of Arizona, Tucson, AZ, United States

**Keywords:** HIV, simian immunodeficiency virus, chimeric antigen receptor, CAR-T cells, CXCR5, B cell follicles, CD8^+^ T cells, HIV cure strategies

## Abstract

There is a need to develop improved methods to treat and potentially cure HIV infection. During chronic HIV infection, replication is concentrated within T follicular helper cells (Tfh) located within B cell follicles, where low levels of virus-specific CTL permit ongoing viral replication. We previously showed that elevated levels of simian immunodeficiency virus (SIV)-specific CTL in B cell follicles are linked to both decreased levels of viral replication in follicles and decreased plasma viral loads. These findings provide the rationale to develop a strategy for targeting follicular viral-producing (Tfh) cells using antiviral chimeric antigen receptor (CAR) T cells co-expressing the follicular homing chemokine receptor CXCR5. We hypothesize that antiviral CAR/CXCR5-expressing T cells, when infused into an SIV-infected animal or an HIV-infected individual, will home to B cell follicles, suppress viral replication, and lead to long-term durable remission of SIV and HIV. To begin to test this hypothesis, we engineered gammaretroviral transduction vectors for co-expression of a bispecific anti-SIV CAR and rhesus macaque CXCR5. Viral suppression by CAR/CXCR5-transduced T cells was measured *in vitro*, and CXCR5-mediated migration was evaluated using both an *in vitro* transwell migration assay, as well as a novel *ex vivo* tissue migration assay. The functionality of the CAR/CXCR5 T cells was demonstrated through their potent suppression of SIV_mac239_ and SIV_E660_ replication in *in vitro* and migration to the ligand CXCL13 *in vitro*, and concentration in B cell follicles in tissues *ex vivo*. These novel antiviral immunotherapy products have the potential to provide long-term durable remission (functional cure) of HIV and SIV infections.

## Introduction

Over 2 million individuals become infected with HIV each year, and nearly 37 million people are currently infected with HIV (1). Current antiretroviral therapy (ART), while effective at reducing viral loads, does not eliminate the virus, thus requiring HIV-infected individuals to remain on ART for life. ART is expensive, inconvenient, demands strict adherence, and, in some cases, leads to drug resistance. In addition, HIV-infected individuals remain at increased risk of cardiovascular disease (2), neurological disease (3), and malignancies (4), and have decreased life expectancies (5). Given these issues and risks, there is great global interest in developing strategies to fully eradicate infectious HIV from the body (“sterilizing cure”), or to achieve durable viral remission in the absence of ART (“functional cure”) (6).

During chronic HIV and simian immunodeficiency virus (SIV) infections prior to the development of AIDS, virus replication is most concentrated within B cell follicles (7–12), primarily within T follicular helper cells (Tfh) (10, 13, 14). Replication is further sustained by infectious virions adhering to the surface of follicular dendritic cells (FDC) *via* antibody and complement complexes in germinal centers (15–19). Although virus-specific CD8^+^ T cells are critical for controlling HIV and SIV infections, they fail to fully suppress viral replication (20). Several mechanisms are thought to contribute to this failure including: the emergence of CTL escape variants (21–28), viral induced MHC class I down-modulation (29, 30), viral latency (31), CTL exhaustion (32–34), and potential Treg inhibition of CTL (35–39). A particularly compelling factor, which we address in this study, is that levels of virus-specific CD8^+^ T cells are low within B cell follicles, thereby permitting ongoing viral replication (8, 9, 40–42).

Migration of cells into the B cell follicle is mediated through the chemokine receptor, CXCR5 (43–45), and its ligand, the chemokine CXCL13 (46, 47), which is expressed by B cells (48–50) and FDCs in follicles (47, 51). We hypothesize that increasing levels of virus-specific CTL in B cell follicles will lead to significantly better control of viral replication in B cell follicles and might lead to sustained remission of HIV infection (42). Several lines of evidence support this hypothesis. In lymphocytic choriomeningitis virus (LCMV)-infected mouse models, adoptive transfer of CXCR5-expressing, LCMV-specific CD8^+^ T cells controlled LCMV infection of Tfh cells and reduced viral loads significantly better than CXCR5^−^ CD8^+^ T cells (52, 53). We previously showed that levels of SIV-specific CTL in lymphoid compartments predicted levels of viral replication in lymphoid compartments (8) and that levels of SIV-specific CTL in follicles tended to predict plasma viral loads (36). Furthermore, it was reported recently that levels of virus-specific CXCR5^+^ cells inversely correlated with viral load in HIV-infected individuals (52). In addition, in a recent SIV CTL vaccine study, it was found that vaccine induced protection from pathogenic SIV challenge was associated with increased levels of CXCR5^+^ virus-specific CD8^+^ T cells (54). Thus, increasing virus-specific CD8^+^ T cells in B cell follicles is predicted to lead to better control of viral replication in lymphoid follicles and decreased viral loads.

In the field of cancer immunotherapy, dramatic successes have been achieved by genetically engineering autologous patient T cells to express a chimeric antigen receptor (CAR). CAR-T cells have shown great promise in treating certain B cell leukemias and lymphomas, and are being actively pursued to treat additional cancers including solid tumors (55–57). Several features make CAR technology particularly appealing in HIV functional cure efforts (58–61). CAR activity is MHC-independent, and thus not compromised by HIV-1 nef-mediated down-modulation of MHC-I in infected cells that facilitates their evasion from conventional cytotoxic T cells (62). The target for an anti-HIV CAR is the viral Env glycoprotein, which is expressed exclusively on infected cells. Env is absolutely essential for virus infectivity and spread, and the targeting motif of the CAR can be designed to recognize strictly conserved Env elements that are refractory to mutational escape. Interestingly, the very first clinical tests of CAR technology were directed against HIV-1 infection, using first-generation CAR constructs employing CD4 as the targeting motif; while minimal virus suppression was achieved, the gammaretroviral-engineered CAR-T were found to be safe, and had stable levels of engraftment with a decay half-life exceeding 16 years (63–66).

Achieving durable HIV/SIV remission in the absence of ART demands long-term persistence of functional CAR-T cells, with minimal chance for virus mutational escape and immune response against the CAR. To this end, we have designed bispecific CARs containing CD4 (domains 1 and 2) linked to a second moiety that binds to a distinct highly conserved site on the HIV-1 Env glycoprotein. The second moiety both enhances CAR potency and prevents the CD4 from acting as an entry receptor in CAR-expressing CD8^+^ T cells (67, 68). In a favored CAR construct (68), the second moiety is the carbohydrate recognition domain of mannose-binding lectin (MBL), which binds to the dense oligomannose patch that is highly conserved on clinically relevant HIV-1 variants. Indeed, compared to a monospecific CD4 CAR, the CD4–MBL CAR displays superior suppressive activity against genetically diverse HIV-1 primary isolates. Immunogenicity concerns are minimized with the CD4–MBL CAR, since both Env-binding components are derived entirely from human protein sequences; moreover, the MBL moiety lacks the equivalent of variable regions that are likely to elicit immune responses during the long-term persistence required to durably maintain HIV suppression.

In the present study, we address another major concern for achieving effective HIV control, namely the likely requirement to enhance CAR-T cell trafficking to B cell follicles. To this end, we engineered rhesus macaque T cells to co-express CXCR5 along with an all-rhesus variant of the CD4–MBL CAR. Results from *in vitro* and *ex vivo* assay systems suggest promising potential of this approach as a means to direct CAR-T cells to B cell follicles, where HIV replication is concentrated.

## Materials and Methods

### Plasmid Constructs and Retroviral Vectors Encoding CARs

All CAR targeting motifs were synthesized by GenScript, codon-optimized for expression in rhesus macaque cells, and subcloned into the plasmid pMSGV1 gammaretrovirus vector backbone (69). The active antiviral CAR employed in this study was a rhesus variant of the human bispecific CAR designated CD4–MBL (68). As a non-reactive negative control, we used the previously described 139 CAR, which does not react with cells in this system. The targeting domains were linked to extracellular hinge, transmembrane and cytoplasmic co-stimulatory domain of rhesus CD8 followed by the activation domain of rhesus CD3 zeta, as previously described (67, 68).

T cells were transduced to express either the rhCD4–MBL CAR, rhCXCR5, or the rhCD4–MBL CAR plus rhCXCR5. For co-expression, bicistronic plasmid constructs (produced by GenScript) were designed in which the rhCD4–MBL gene was linked to the downstream rhCXCR5 gene. CXCR5 expression was driven by either the ECMV internal ribosome entry site (IRES) or the self-cleaving P2A peptide from porcine teschovirus-1 with a GSG linker added at the N-terminus of the P2A peptide sequence (70). The corresponding gammaretroviruses were generated for expression of these genes in rhesus macaque T cells. In most experiments, these plasmids were co-transfected with the plasmid pBS-CMV-gagpol (71) (a gift from Dr. Patrick Salmon, Addgene plasmid #35614), a plasmid encoding RD114 envelope glycoprotein (72), and the plasmid pMD.G encoding VSV-G envelope (73) (a gift from Dr. Scott McIvor) at ratios of 3:1:1:0.4, respectively. Retroviral vector supernatants were collected 48 h after transfection, and were titrated by transducing HEK293T cells. Retrovirus was snap frozen and stored at −80°C. In the SIV suppression studies, gammaretrovirus vector production was carried out as previously described (67).

### Transduction of Rhesus T Cells

Primary rhesus macaque PBMC, or CD8^+^ T cells enriched by negative selection (Miltenyi), were activated for 2 to 3 days in six-well plates with plate-bound anti-CD3 (FN18) and soluble anti-CD28.2 (both from NHP Reagent Resource) in either RPMI supplemented with 10% heat inactivated FBS, 100 U/ml penicillin, 100 µg/ml streptomycin, and 300 IU/ml IL-2, for early experiments, or in X-Vivo 15 completed with 10% heat inactivated FBS, 100 U/ml penicillin, 100 µg/ml streptomycin, 2 mM glutamine, and 50 IU/ml IL-2 for later experiments. RetroNectin (TaKaRa)-mediated transduction was carried out on the activated T cells. Retroviral vector supernatants, diluted in serum-free media, were added (eventual MOI of 0.5) to RetroNectin-coated six-well plates and centrifuged for 2 h at 2,000 × *g* to facilitate binding of the retrovirus. After removal of the unbound retrovirus, activated PBMC or CD8^+^ T cells (1.5 × 10^6^ cells/well) were added to the wells and centrifuged at 1,000 × *g* for 10 min. Mock-transduced cells were subjected to exactly the same procedures without the addition of retrovirus to the RetroNectin-coated wells. Cells were cultivated in the media listed above for 5–6 days prior to analysis by flow cytometry.

### Flow Cytometry

Cells were analyzed using an LSR Fortessa flow cytometer (BD Bioscience). The following antibodies were used: CD4 (M-T477, reactive with endogenous rhCD4 and the rhCD4–MBL CAR), CD3 (SP34-2), CD8 (RPA-T8) (all from BD Bioscience), CXCR5 (MU5UBEE) (eBioscience), MBL2 (3E7) (Invitrogen). Viability was assessed with the Live/Dead Fixable Near IR Dead Cell Stain Kit (Invitrogen). A minimum of 70,000 events were acquired for each sample. Data analysis utilized FlowJo v10 (FlowJo, LLC).

### *In Vitro* Transwell Migration Assay

Rhesus macaque PBMCs were transduced with the CAR or CAR/CXCR5 vectors, or mock-transduced. Samples were run in duplicate. For each sample, one million cells in 100 µl X-Vivo-15 media containing 0.1% BSA were placed in the upper chamber of a 24-well plate, with a 5.0-µm transwell membrane (Costar). To the lower chamber containing 600 µl X-Vivo 15 and 0.1% BSA, either CXCL12 at 1 µg/ml or CXCL13 at 2.5 µg/ml (both from ProSpec) were added. No chemokine was added to control wells. After incubation for 4 h at 37°C, cells were collected from the lower chamber, fixed with 1% paraformaldehyde, and counted on a Cytoflex flow cytometer (Beckman). All samples were normalized with the addition of AccuCheck Counting Beads (Invitrogen). Specific cell migration was determined by first subtracting the number of cells that migrated to media alone from the number of cells that migrated to the chemokine and then dividing by the number of cells added to the upper chamber.

### *Ex Vivo* B Cell Follicle Migration Assay

Chimeric antigen receptor- and CAR/CXCR5-transduced rhesus CD8^+^ T cells were used in conjunction with fresh lymph node tissue sections from allogeneic rhesus macaques. A gelatin sponge (7 mm Gel foam by Pfizer) was cut to fit and placed into a six-well plate containing 3–4 ml of RPMI with 20% heat inactivated FBS. The sponge was hydrated for 1 h at 37°C. Fresh rhesus macaque lymph nodes, collected at the Wisconsin National Primate Research Center, were shipped in chilled RPMI containing 100 µg/ml heparin overnight on ice blocks. Lymph nodes were cut into 0.5 cm × 0.5 cm pieces and embedded in 40°C PBS-buffered 4% low-melt agarose and cut into 300-µm thick slices using a Compresstome, as we have previously described (74). Tissue sections and associated agarose were laid flat on the hydrated sponge without being submerged. Transduced CD8^+^ T cells were stained with a 5-µM solution of Cell Trace Violet Dye (CTV) (Molecular Probes). The dye was added at a 1:1 ratio to 1 × 10^7^ cells/ml suspended in PBS/10% FBS, and cells were incubated for 15 min at 37°C, followed by two washes with complete RPMI supplemented with 10% heat inactivated FBS 100 U/ml penicillin, and 100 µg/ml streptomycin. For each fresh tissue section, one million CTV-stained transduced CD8^+^ T cells were re-suspended in 20–30 µl complete RPMI and were slowly pipetted onto the surface of the tissue. Tissue sections were incubated at 37°C for 6 h followed fixation with 4% PBS-buffered paraformaldehyde for 2 h at RT. After fixation, sections were washed with chilled PBS containing 100 µg/ml heparin (PBS-H). Antigen retrieval was carried out by boiling tissues 3× in 0.01 M urea for 30 s. Tissues were permeabilized and blocked with PBS-H containing 0.3% Triton x-100 and 2% normal goat serum for 1 h, then incubated overnight with mouse-anti-human CD20 (0.19 µg/ml, clone L26, Novocastra) to label B cells and rat-anti-human CD3 (2 µg/ml, CD3-12, Bio-Rad) to label T cells. After washing with PBS-H, secondary antibody staining was carried out by incubating tissues overnight with goat-anti-mouse-IgG/Alexa 488 (0.75 µg/ml Jackson ImmunoResearch Laboratories) and goat-anti-rat-IgG/Cy5 (0.3 µg/ml, Jackson ImmunoResearch Laboratories). All incubations were done at 4°C on a rocking platform. Sections were imaged using a Leica confocal microscope. 512 × 512 pixel *z*-series were collected using a step size of 2 µm and with collection initiated at least 50 µm deep into each section. B cell follicles were identified morphologically as clusters of brightly stained closely aggregated CD20^+^ cells. Areas that showed loosely aggregated B cells that were ambiguous as to whether the area was a follicle were not included. Cell counts were done with individual *z*-scans. The total number of CTV-stained cells was counted inside follicles and the adjacent area outside of the follicles. For each sample, 2–3 tissue sections and a minimum of three follicles (range 3–8) were evaluated.

### SIV Suppression Assay

To generate SIV-infected target cells, rhesus macaque PBMCs were re-suspended at 5 × 10^5^/ml in complete medium, transferred to a T25 flask, and incubated at 37°C in 5% CO_2_ for 2–3 days. The PBMCs were washed, adjusted to 3 × 10^6^/ml in total of 4 ml volume in complete media containing 30 IU/ml IL-2, and incubated with 200–600 TCID_50_/ml of virus for 24 h at 37°C in 5% CO_2_. Infected cells were washed three times using 20 ml of medium per wash and then re-suspended in complete medium at a density of 1.5 × 10^6^ cells per ml in 96-well round bottom plates. To generate effector cells, T cells (derived from activated PBMCs) were transduced with the indicated gammaretroviral vectors. In triplicate, 100 µl of SIV-infected targets were mixed with 100 µl of serially diluted effectors. Cocultures were incubated at 37°C in 5% CO_2_ for a total of 16 days. On the indicated days, supernatants were collected, and p27 content was determined by ELISA (ABL, Inc.).

### Statistical Analysis

All statistical analyses assumed two-sided tests with *P* < 0.05 considered significant. Paired *t*-tests with pooled variance were used to evaluate co-expression levels of the CAR and CXCR5 *via* IRES versus P2A constructs An unpaired *t*-test with pooled variance was used to evaluate groups in the CXCL12 *in vitro* migration assays while an unpaired Welch’s *t*-test of unequal variance was used to evaluate groups in the CXCL13 *in vitro* migration assays. Paired *t*-tests with pooled variance were used in all statistical analyses in the *ex vivo* migration assay. The F:EF ratios were log transformed before analysis. Statistical analyses were conducted using GraphPad Prism (Version 6.01; GraphPad Software, Inc., La Jolla, CA, USA).

## Results

The goal of this study was to engineer rhesus macaque T cells to co-express a potent anti-SIV CAR along with CXCR5, in order to promote CAR-T cell trafficking to B cell follicles. To this end, we designed constructs for expression of the CARs, without or with co-expression of CXCR5. CAR-transduced T cells were analyzed using both an *in vitro* transwell assay of chemokine-directed cell migration and a novel *ex vivo* B cell follicle migration assay. In addition, we tested the ability of T cells expressing CAR and CXCR5 to suppress viral replication *in vitro*.

### CAR and CXCR5 Expression in Transduced Primary Rhesus Macaque T Cells

**Figure 1:**
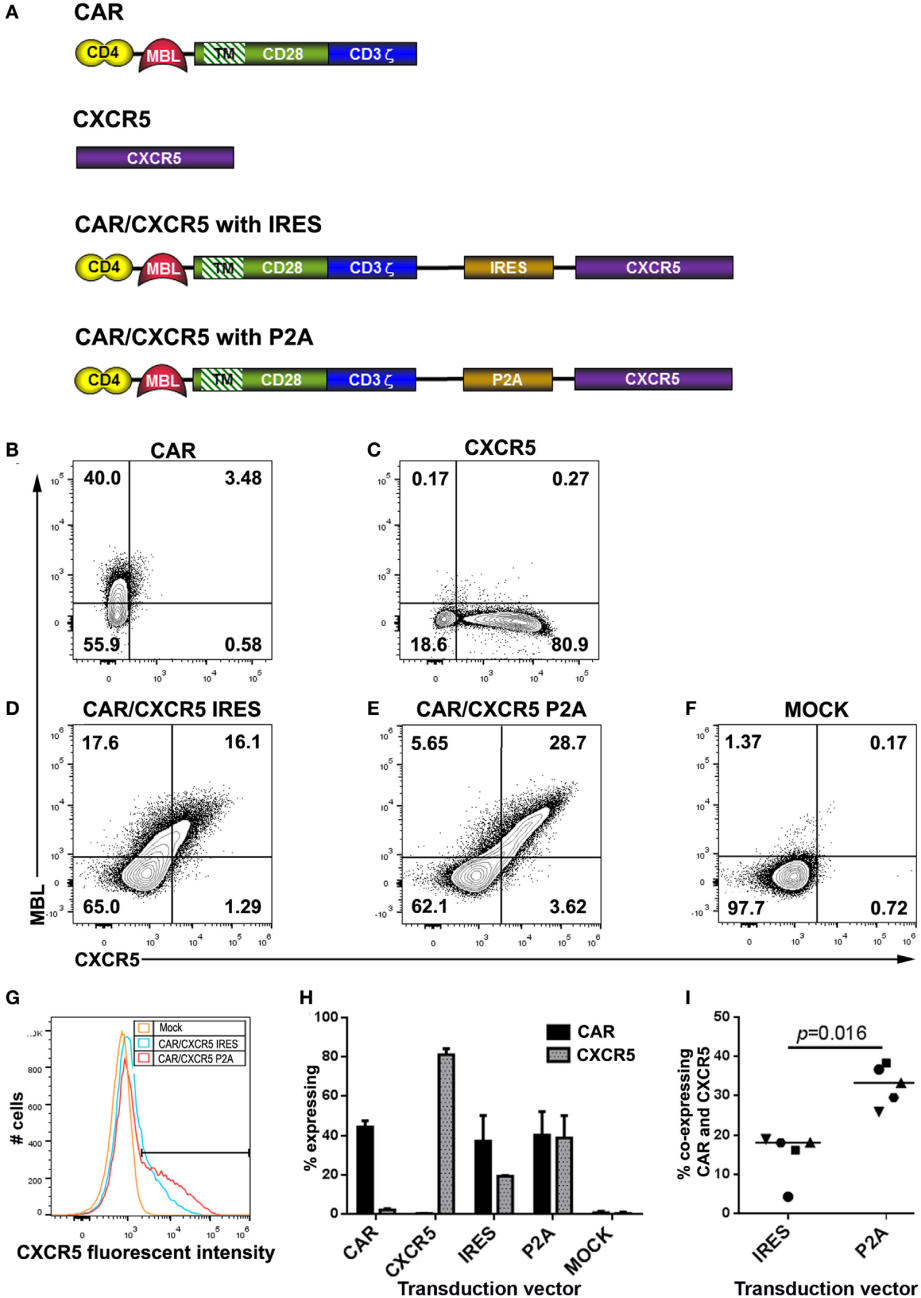
Construct design, and expression in rhesus macaque T cells. **(A)** Schematic figures showing constructs encoding the chimeric antigen receptor (CAR) (rhCD4–MBL CAR), rhCXCR5, and the bicistronic CAR/CXCR5 encoding both proteins, with CXCR5 expression mediated by IRES or P2A. In all cases, the targeting domains are linked to domains from rhesus CD28 including a short extracellular hinge, transmembrane TM, and cytoplasmic signaling, followed by the CD3 activation domain. Cells were transduced with gammaretroviral vectors encoding **(B)** CAR, **(C)** CXCR5, **(D)** CAR/CXCR5 (IRES), **(E)** CAR/CXCR5 (P2A), or **(F)** mock-transfected, and analyzed by flow cytometry. Cells were pre-gated sequentially on lymphocytes, singlets, live cells, and CD3^+^ cells (T cells) and evaluated for CAR and CXCR5 expression, using antibodies against mannose binding lectin (MBL) and CXCR5, respectively. **(G)** Histogram depicting fluoroscent intensites of CXCR5 expression from samples shown in panels **(D–F)**. **(H)** Median percentage of T cells that expressed the CAR and CXCR5 in activated PBMCs transduced with CAR (*n* = 3), CXCR5 (*n* = 3), CAR/CXCR5 (IRES) (*n* = 5), CAR/CXCR5 (P2A) (*n* = 5) and mock-transduced (*n* = 5). **(I)** The percentage of T cells that co-expressed the CAR and CXCR5 in activated PBMCs transduced with either CAR/CXCR5 (IRES) or CAR/CXCR5 (P2A).

For this study, we developed gammaretroviral vectors encoding the rhCD4–MBL CAR and rhCXCR5, and vectors encoding bicistronic constructs to express both proteins. We developed two variations of bicistronic vectors, one with CXCR5 co-expression driven by an internal ribosome entry site (IRES) and the other *via* a P2A self-cleavage site (70). For simplicity, the constructs encoding the rhCD4–MBL CAR alone or the bicistronic rhCD4–MBL CAR plus rhCXCR5 are, respectively, referred to as CAR or CAR/CXCR5; for the latter, the use of either the IRES or P2A modalities is indicated. The constructs are shown schematically in Figure 1A.

In Figures 1B–F, T cells derived from activated rhesus PBMCs were transduced with gammaretroviral vectors encoding the CAR or CXCR5 genes alone, or the bicistronic CAR/CXCR5 constructs (IRES or P2A). Cell viabilities posttransduction were 87–90% (data not shown). Antibodies directed against MBL or CXCR5 were used to detect surface expression of the CAR and CXCR5, respectively. Transduction with the CAR (Figure 1B) or CXCR5 (Figure 1C) vectors gave the expected surface expression of the corresponding individual proteins. For vectors encoding the bicistronic CAR/CXCR5, the P2A-based construct yielded a clear population of cells expressing both CAR and CXCR5, with only a small fraction of cells expressing only one of the proteins; by contrast, the IRES-based construct appeared less effective at co-expressing CXCR5 relative to CAR, since the fraction of cells expressing only the CAR was comparable to that expressing both proteins, with a minimal fraction expressing only CXCR5 only (Figures 1D,E). These results are consistent with the efficient P2A system producing equivalent amounts of the two post-cleavage components of a bicistronic construct, as contrasted with the relatively inefficient expression of the downstream component in the IRES system (75, 76). Moreover, as indicated in Figure 1G, the P2A-based construct produced cells with nearly twofold higher surface expression levels of CXCR5 than obtained with the IRES-based construct (median 1.8-fold higher; range 1.4- to 2.2-fold).

The percentages of T cells that expressed the CAR and CXCR5 with each construct are shown in Figure 1H. Transduction with the vectors encoding CAR-only or CXCR5-only yielded a median of 44.4% (range 40–47.6%) and 81.1% (range 51.8–84.2%) of cells expressing each protein, respectively. Cells transduced with the IRES-based bicistronic CAR/CXCR5 vector showed higher number of cells expressing the CAR compared to CXCR5, with a median cell expression of 37.2% (range 5.6–50.2%) for the CAR and 19.4% (range 4.7–19.7%) for CXCR5. In contrast, cells transduced with the P2A-based CAR/CXCR5 vector showed similar expression of the two proteins, with a median of 40.2% (range 37.3–52.2%) for the CAR and 38.9% (range 27.5–50.2%) for CXCR5. Similar transduction efficiencies were found with enriched rhesus CD8 T cells transduced with these vectors (data not shown). The percentage of cells that co-expressed CAR and CXCR5 is shown in Figure 1I. Cells transduced with the IRES-based construct showed a median co-expression efficiency of 18.1% (range 4.3–18.9%), whereas cells transduced with the P2A-based construct resulted in a significantly higher co-expression efficiency of 33.3% (range of 25.9–38.3). Thus, the data in Figure 1 establish the suitability of the P2A-based bicistronic system for efficient co-expression of CAR and the B cell follicle-homing chemokine receptor CXCR5, and its superiority over the IRES-based system.

### CXCR5 Co-Expression Promotes CAR-T Cell Migration Selectively to CXCL13 *In Vitro*

**Figure 2:**
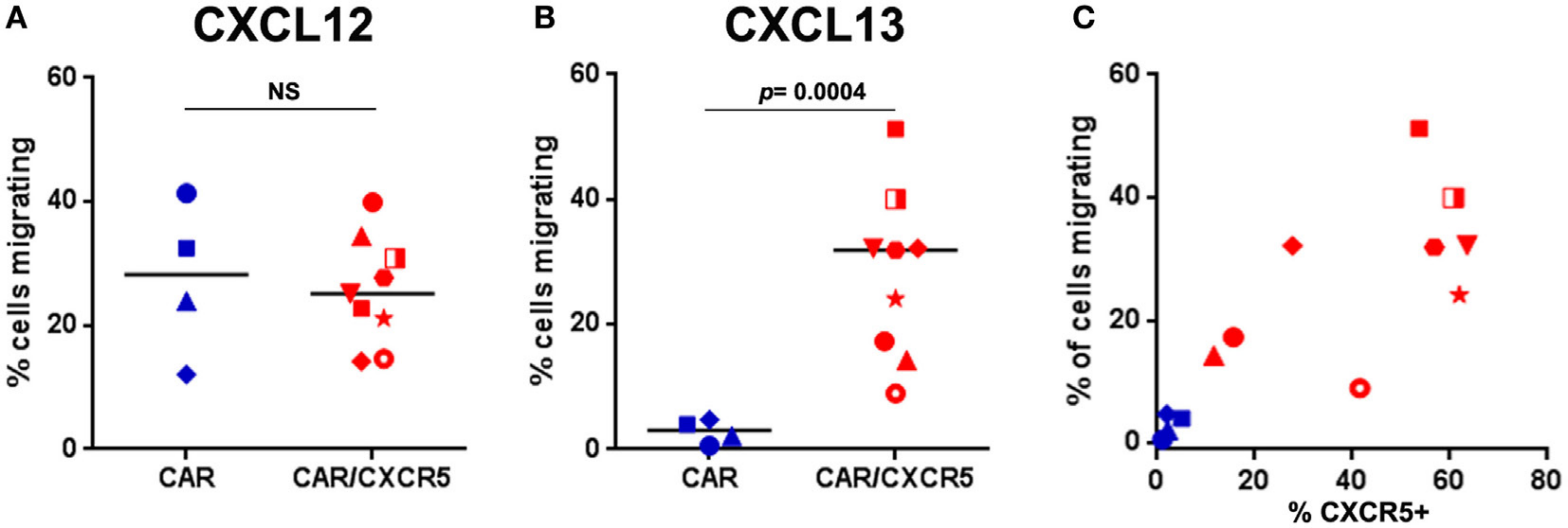
Co-expression of CXCR5 promotes selective migration of chimeric antigen receptor (CAR)-T cells toward CXCL13 *in vitro*. The percentage of CAR- or CAR/CXCR5-transduced PBMC that migrated toward **(A)** CXCL12 (SDF-1) or **(B)** CXCL13 was measured in transwell plates. **(C)** The relationship between the percentage of cells expressing CXCR5 and the percentage of cells that migrated. In all panels, each data point symbol represents the mean value of duplicate samples obtained with cells from individual animals, with colors indicating transduction with CAR (blue) or CAR/CXCR5 (red).

We next tested the ability of CXCR5 co-expression to promote migration of CAR-T cells toward CXCL13, the chemokine ligand for CXCR5. To this end, we utilized an *in vitro* transwell migration assay. Using this assay, we found that both CAR-transduced and CAR/CXCR5-transduced PBMCs similarly migrated toward a positive control chemokine CXCL12 (SDF-1α) that is strongly chemotactic for lymphocytes (77) demonstrating the ability of both CAR and CAR/CXCR5-transduced cells to migrate to a chemotactic stimulus (Figure 2A). In contrast, significantly more CAR/CXCR5-transduced than CAR-transduced PBMCs migrated toward CXCL13 (Figure 2B). Furthermore, increasing specific migration to CXCL13 was seen with an increase in the percentage of cells expressing CXCR5 (Figure 2C). For these studies, a median of 54% (range 12–64%) of CAR/CXCR5-transduced cells expressed CXCR5. By contrast, a median of only 2% (range 1–5%) of the CAR-transduced cells expressed CXCR5 and they showed minimal migration to the stimulus. These results demonstrate that co-expression of CXCR5 promotes selective migration of the CAR-T cells toward CXCL13 *in vitro*.

### CAR/CXCR5-Transduced CD8^+^ T Cells Selectively Migrate into B Cell Follicles *Ex Vivo*

**Figure 3:**
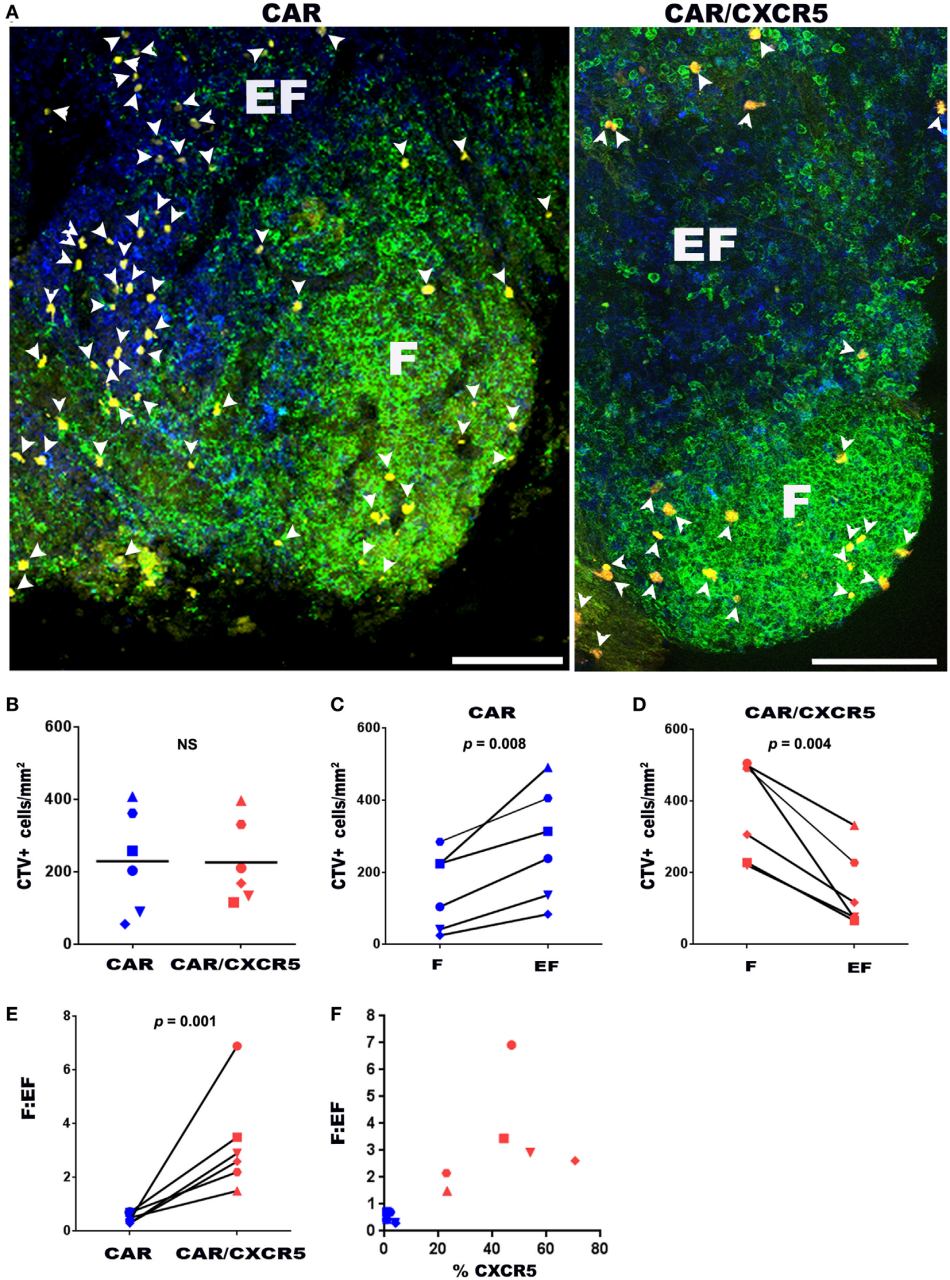
*CXCR5* co-expression enhances CD8^+^ CAR-T cell migration to B cell follicles *ex vivo*. **(A)** Chimeric antigen receptor (CAR) or CAR/CXCR5-transduced rhesus macaque CD8^+^ T cells were stained with cell trace violet dye (CTV) (pseudo-colored yellow), then pipetted on to fresh rhesus macaque lymph node sections and incubated for 6 h at 37°C. Sections were then fixed and stained with anti-CD20 antibodies (green) to delineate B cell follicles (F) and anti-CD3 antibodies (blue) to delineate the T cell zone and extrafollicular areas (EF). Arrowheads indicate CTV^+^ cells. Confocal images were collected with a 20× objective. Scale bars equal 100 µm. **(B)** Similar total levels of CFSE-labeled CD8^+^ T cells were detected in tissues incubated with CAR- and CAR/CXCR5- transduced cells. **(C)** CAR-transduced cells showed higher levels in the extrafollicular regions than in the follicles. **(D)** By contrast, CAR/CXCR5-transduced cells showed increased levels within B cell follicles. **(E)** CAR/CXCR5-transduced cells showed higher F:EF ratios compared to CAR-transduced cells. **(F)** The relationship between the percentage of transduced cells that expressed CXCR5 and F:EF ratios. Each symbol represents individual animals from which CD8^+^ T cells were derived.

As an additional means to evaluate the ability of CXCR5 to promote selective migration of CAR-T cells, we developed a novel *ex vivo* B cell follicle migration assay. This method was adapted from previously described *ex vivo* live tissue migration assays that tracked T cells in mouse thymus tissue using two-photon microscopy (78, 79). For these studies, we evaluated the migration of CTV-labeled CAR- and CAR/CXCR5-transduced primary rhesus macaque CD8^+^ T cells in fresh lymph node tissue sections. Figure 3A shows representative images of sections incubated with CTV-labeled CAR and CAR/CXCR5-transduced cells. Similar levels of total CTV^+^ cells were detected in lymph node sections incubated with CAR versus CAR/CXCR5-transduced cells (Figure 3B). While total numbers of cells were similar, significant differences were observed in the levels of CAR- compared to CAR/CXCR5-transduced cells in follicular and extrafollicular compartments. Significantly lower levels of CTV^+^ cells were found in follicular compared to extrafollicular areas in sections incubated with CAR-transduced cells (Figure 3C). In contrast, significantly higher levels of CTV^+^ cells were found in follicular compared to extrafollicular areas in sections incubated with CAR/CXCR5-transduced cells (Figure 3D). As a result, significantly large increases in the follicular to extrafollicular ratios (F:EF) of CTV-labeled cells were detected in the tissue sections incubated with CAR/CXCR5- compared to CAR-transduced T cells. Sections incubated with CAR/CXCR5-transduced cells showed a median F:EF ratio of 2.8 (range of 1.5–6.9), whereas sections incubated with CAR-transduced T cells showed a median ratio of 0.4 (range 0.3–0.7) (Figure 3E). An increased follicular to extrafollicular ratio was seen with an increase in the percentage of cells expressing CXCR5 (Figure 3F). A median of 46% (range 23–71%) of CAR/CXCR5-transduced cells expressed CXCR5 and they showed relatively high F:EF ratios. By contrast, a median of only 1.6% (range 0.2–4.1%) of the CAR-transduced cells expressed CXCR5 and they showed correspondingly low F:EF ratios. Thus, in this novel *ex vivo* B cell follicle migration assay, CAR/CXCR5- but not CAR-transduced CD8^+^ T cells preferentially migrated to B cell follicles.

### CXCR5 Co-Expression Does Not Impair CAR-T Cell-Mediated Suppression of SIV Replication *In Vitro*

**Figure 4:**
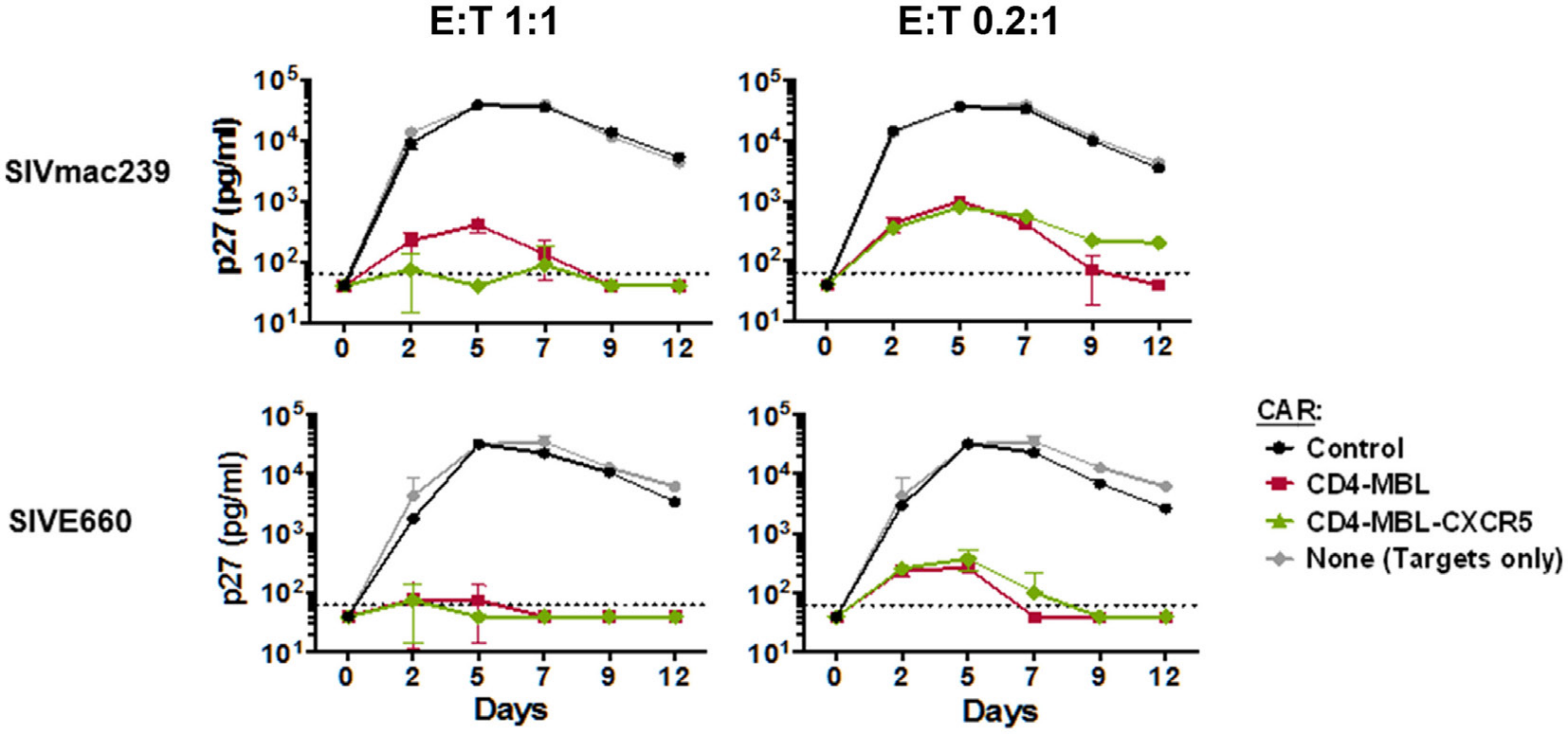
Chimeric antigen receptor (CAR)/CXCR5-transduced T cells suppress simian immunodeficiency virus (SIV) *in vitro*. PBMC target cells were infected with the indicated SIV_mac239_ and SIV_E660_ isolates for 24 h. The cells were then washed and mixed with the effector cells transduced as indicated, at effector-to-target ratios (E:T) of 1:1 (left panels) or 0.2:1 (right panels). Culture supernatants were collected on the indicated days, and the presence of virus was determined by p27 ELISA. The effector cells were transduced either with the CD4–mannose-binding lectin (MBL)–CAR alone or CD4–MBL–CAR plus CXCR5. As negative controls, no effector cells, or cells transduced with the negative control 139 CAR were used.

The all-rhesus CD4–MBL CAR (rhCD4–MBL) displayed potent suppression of multiple SIV strains (Hajduczki et al., manuscript in preparation). For this study, we tested whether co-expression of CXCR5 affected the potency of SIV suppression by T cells expressing the rhesus CD4–MBL CAR. PBMCs transduced with the CAR or CAR/CXCR5 vectors, were cocultured with rhesus PBMC targets infected with two different pathogenic SIV isolates, SIV_mac239_ and SIV_E660_. The negative controls employed included adding no effector T cells, and adding effector T cells that were transduced with the 139 CAR that recognizes an irrelevant epitope [a glioma-specific variant of the epidermal growth factor receptor (80)]. Robust spreading of viral infection by both SIV strains was evident in the presence of the negative control effector cells (no effector T cells and 139 CAR-transduced T cells). In contrast, CAR-transduced and CAR/CXCR5-transduced effectors suppressed infection by both strains with equivalent high potency over the 12-day infection, at E:T ratios of 1:1 or 0.2:1 (Figure 4). These data demonstrate that the antiviral activity of CAR-T cells is not altered by co-expression of the CXCR5 follicular trafficking chemokine receptor on the effector cell surface.

## Discussion

In most HIV-infected individuals and SIV-infected rhesus macaques, virus-specific CTL fail to accumulate to high levels in B cell follicles (8, 9, 40–42), where virus replication is most concentrated prior to the development of AIDS (7–12). The paucity of virus-specific CTL in follicles permit ongoing replication (8, 36, 52, 54). We hypothesize that increasing levels of virus-specific CTL in follicles will lead to better control of viral replication and may lead to long-term durable remission in the absence of ART, i.e., a “functional cure.” In this study, we developed tools to test this hypothesis in the SIV-infected rhesus macaque model of HIV infection. To this end, we developed gammaretroviral vectors for co-expression of a potent bispecific anti-SIV CAR (rhCD4–MBL) and the B cell follicle-homing chemokine receptor CXCR5.

Our initial bicistronic constructs encoding both the CAR and CXCR5 utilized an internal ribosome entry site (IRES) to achieve co-expression. We found inconsistent and often low levels of cells that co-expressed both the CAR and CXCR5. This was likely due to inefficient initiation of translation at the IRES. This finding was not altogether surprising as it is well known that IRES-dependent gene expression from a bicistronic construct is not always efficient in all cell systems (75, 76). To achieve more consistent levels of CAR and CXCR5 co-expression, we tested an alternative construct with the porcine teschovirus-1 P2A self-cleavage site between the CAR and CXCR5 genes. The P2A-sequence allows the cell to produce both proteins without re-initiation of translation due to a “stop and go” translational effect mediated by the ribosome, thereby resulting in similar levels of expression of the two proteins (70, 81). In contrast with the IRES-based CAR/CXCR5 construct, T cells transduced with the P2A-based construct consistently produced efficient co-expression of both proteins on the T cell surface. Moreover, the P2A yielded nearly twofold higher levels of CXCR5 at the cell surface. These results highlight the superiority of the P2A compared to the IRES modality for advancing the CAR/CXCR5 system as an immunotherapy product.

Using the P2A system, we demonstrated CXCR5 functionality in promoting targeted migration of CAR-T cells. In an *in vitro* migration assay, CXCR5 co-expression drove selective migration of rhesus CAR-T cells toward CXCL13, the chemokine ligand for CXCR5 responsible for follicular homing. Moreover, using in a novel *ex vivo* B cell follicle migration assay, we demonstrated that CXCR5 co-expression promoted accumulation of rhesus CAR-T cells in B cell follicles of rhesus lymphoid tissue. This finding is supported by the recent report showing that rhesus CD8^+^ T cells engineered to express human CXCR5 and infused into rhesus macaques accumulated within B cell follicles *in vivo* (82). The T cells used in that study, however, did not contain a viral-targeting CAR or other antiviral moiety required for suppressing virus replication.

We previously demonstrated that human CD4–MBL CAR-T cells are capable of potently suppressing *in vitro* replication of genetically diverse HIV-1 isolates (68). The rhesus variant of this CAR displays potent suppressive activity against multiple SIV strains (Hajduczki et al., in preparation). Here, we show that co-expression of rhesus CXCR5 causes no impairment of the SIV-suppressive activity of this CAR.

As mentioned, we hypothesize that treatment with autologous CAR/CXCR5-transduced T cells can be a valuable component for achieving sustained remission of HIV. Future studies to evaluate the *in vivo* efficacy of CAR/CXCR5 immunotherapy must address multiple complexities under active study in the cancer field (56, 83, 84), plus others distinct for HIV (60, 85, 86). Robust proliferation and persistence of the adoptively transferred cells is especially critical for the long-term (life-long?) viral suppression required for an HIV functional cure. Diverse aspects are being investigated, including choice of optimal cell type (T cells early stages of differentiation, hematopoietic stem cells, etc.), mode of *ex vivo* cell expansion, requirements for CAR expression on both CD4^+^ and CD8^+^ T cells, alternative methods for CAR gene introduction (viral vector transduction, targeted gene insertion), influence of alternative co-stimulatory domains (CD28, 4-1BB, etc.), and strategies to limit CAR-T cell exhaustion.

Additional challenges confront CAR-based immunotherapy against HIV. A particular concern involves the potential for the CAR-T cells to become infected, which would likely compromise their function and persistence. The bispecific CD4-based CARs such as CD4–MBL (67, 68) are advantageous in that the second moiety prevents the CD4 from acting as an HIV entry receptor on CAR-expressing CD8^+^ T cells; however, an additional mode of protection is required for CAR-expressing CD4^+^ T cells, which are susceptible to infection *via* the endogenous CD4 molecules. Another issue is the requirement for antigenic stimulation to maintain the CAR-T cells. Within the B cell follicle, infected Tfh cells may provide the necessary stimulatory activity. If CAR-T cells are administered after effective HIV suppression with ART, the required antigenic stimulation presumably would occur upon drug cessation. For maintenance of CAR-mediated suppression in the absence of ART, the spontaneous activation of latently infected cells may provide the necessary antigenic stimulation. Well-designed studies in suitable animal models will help pave the way toward efficacious CAR-T cell therapy as a component of an HIV functional cure.

## Ethics Statement

Indian-derived rhesus macaque monkeys (*Macacca mulatta*) described in this study were housed at the Wisconsin National Primate Reasearch Center in accordance with the regulations of the American Association of Accreditation of Laboratory Animal Care and the standards of the Association for Assessment and Accreditation of Laboratory Animal Care International. All protocols and procedures were approved by the relevant Institutional Animal Care and Use Committee at the University of Wisconsin-Madison. All animals were housed indoors in an SOP-driven, AAALAC-accredited facility. Husbandry and care met the guidance of the Animal Welfare Regulations, OLAW reporting and the standards set forth in The Guide for the Care and Use of Laboratory Animals.

## Author Contributions

KH assisted with vector construction, performed transduction experiments, performed the *in vitro* migration assays, and assisted with drafting this manuscript; AH performed the viral suppression assays and assisted with drafting of this manuscript, MP optimized protocols, oversaw and performed virus production and transduction experiments in the Skinner lab, assisted with subcloning genes and sequencing vectors, and drafted this manuscript; GM developed and performed the *ex vivo* migration assays; DV-I constructed and characterized CAR constructs; ER provided study oversight, assisted with flow cytometry, and oversaw acquisition and isolation of primate cells; EC provided study oversight, obtained funding, and assisted with drafting this manuscript; EB oversaw all aspects of CAR design and functional characterization viral suppression assays, obtained funding, and contributed to drafting this manuscript; PS conceived of the studies, obtained funding, provided study oversight, and drafted this manuscript.

## Conflict of Interest Statement

EB is co-inventor on patent applications for CD4-based bispecific CARs, owned by the National Institutes of Health. PS is inventor on a provisional patent application for CAR/CXCR5 immunotherapy, owned by the University of Minnesota. All other authors declare that the research was conducted in the absence of any commercial or financial relationships that could be construed as a potential conflict of interest.
